# Metabolic and diffusional limitations of photosynthesis in fluctuating irradiance in *Arabidopsis thaliana*

**DOI:** 10.1038/srep31252

**Published:** 2016-08-09

**Authors:** Elias Kaiser, Alejandro Morales, Jeremy Harbinson, Ep Heuvelink, Aina E. Prinzenberg, Leo F. M. Marcelis

**Affiliations:** 1 Horticulture and Product Physiology Group, Department of Plant Sciences, Wageningen University, PO Box 16, 6700 AA Wageningen, The Netherlands; 2 Centre for Crop Systems Analysis, Department of Plant Sciences, Wageningen University, PO Box 430, 6700 AK Wageningen, The Netherlands; 3 Laboratory of Genetics, Department of Plant Sciences, Wageningen University, PO Box 16, 6700 AA Wageningen, The Netherlands

## Abstract

A better understanding of the metabolic and diffusional limitations of photosynthesis in fluctuating irradiance can help identify targets for improving crop yields. We used different genotypes of *Arabidopsis thaliana* to characterise the importance of Rubisco activase (Rca), stomatal conductance (g_s_), non-photochemical quenching of chlorophyll fluorescence (NPQ) and sucrose phosphate synthase (SPS) on photosynthesis in fluctuating irradiance. Leaf gas exchange and chlorophyll fluorescence were measured in leaves exposed to stepwise increases and decreases in irradiance. *rwt43*, which has a constitutively active Rubisco enzyme in different irradiance intensities (except in darkness), showed faster increases than the wildtype, Colombia-0, in photosynthesis rates after step increases in irradiance. *rca-2*, having decreased Rca concentration, showed lower rates of increase. In *aba2-1*, high g_s_ increased the rate of change after stepwise irradiance increases, while in C24, low g_s_ tended to decrease it. Differences in rates of change between Colombia-0 and plants with low levels of NPQ (*npq1-2*, *npq4-1*) or SPS (*spsa1*) were negligible. In Colombia-0, the regulation of Rubisco activation and of g_s_ were therefore limiting for photosynthesis in fluctuating irradiance, while levels of NPQ or SPS were not. This suggests Rca and g_s_ as targets for improvement of photosynthesis of plants in fluctuating irradiance.

In physiological research, plants are often studied under constant environmental conditions. However, plants grow in a variable environment, with changes occurring in the time range of seconds or less^1^. Of the factors important for net photosynthesis (A_n_), irradiance changes most quickly^2^, causing a lag between changes in irradiance and changes in A_n_, due to the slower regulation of photosynthesis^3^. This lag decreases light-use efficiency relative to the steady state and transiently increases excess irradiance, possibly harming the photosynthetic apparatus^4^. Leaves engage various mechanisms in response to fluctuating irradiance. Among the best known mechanisms are the regulation of enzymes of carbon fixation and sucrose metabolism, non-photochemical energy dissipation and stomatal conductance (g_s_^3^,^5^). Although difficult to measure, cyclic electron transport may be another important mechanism (recently reviewed by Yamori and Shikanai^6^), due to a potential regulatory role and the balance of ATP versus NADPH production. During induction of photosynthesis in leaves adapted to darkness or low irradiance, the slow regeneration of ribulose-1,5-bisphosphate (RuBP) is typically most limiting until 60 seconds after illumination^7^. Thereafter, both the slow carboxylation due to partially inactive Rubisco (time to full activation: ~10 minutes) and slow stomatal opening (10–60 minutes) can limit the rate at which photosynthesis increases^8^. Thus, the slow rate of change of these mechanisms results in the lag between changes in irradiance and A_n_ and the resulting reduction of plant productivity^9^. Reductions in assimilation due to these physiological limitations can be up to 35% per day (subject to light environment and genotype^10^), and understanding them better may pave the road towards higher yields^11^,^12^.

Our understanding of the metabolic constraints of photosynthesis in fluctuating irradiance (hereafter: ‘dynamic photosynthesis’) have mainly come from biochemical studies^7^,^13^,^14^, with less use being made of genetic diversity. Naturally occurring ecotypes, mutations, cultivars and genetically modified accessions offer a range of genotypes with specific properties, that could be used to study dynamic photosynthesis^5^. *Arabidopsis thaliana* possesses a wide, well documented genotypic diversity, which has been extended by selecting for mutations and by transgenic modifications.

Rubisco catalyses CO_2_ assimilation and its activation limits A_n_ after irradiance increases^13^,^15^. In the chloroplast stroma, several inhibitory compounds are present and bind to Rubisco. To maintain sufficient Rubisco activity, these inhibitors must be removed from the active sites by the ATPase Rubisco activase (Rca^16^). In *Arabidopsis thaliana*, there are two isoforms of Rca, the larger α-isoform and the smaller β-isoform^17^. In plants containing both isoforms, redox-regulation of the α-isoform affects the ADP sensitivity of the holoenzyme (composed of both isoforms^18^,^19^). In low irradiance (i.e. high ADP/ATP ratio), the α-isoform is less active and the rate of overall Rubisco activation is low. Since Rca is a central regulator of Rubisco activity, how these isoforms, or their concentration affect dynamic photosynthesis is an important yet unresolved question.

After CO_2_ assimilation by Rubisco, a fraction of the triose phosphates leaves the chloroplast in exchange for orthophosphate (P_i_) from the cytosol. In the cytosol, triose phosphate is converted to sucrose, and sucrose phosphate synthase (SPS) plays a central role in this pathway^20^. In certain circumstances, such as photosynthetic induction in saturating CO_2_, irradiance-dependent activation of SPS can be slower than that of Calvin cycle enzymes, making the Calvin cycle transiently P_i_-limited^14^. Furthermore, after irradiance decreases, an overshoot in sucrose synthesis can transiently drain metabolites from the Calvin cycle, transiently decreasing A_n_^21^. Plants with reduced SPS concentration may therefore exhibit slower increases in A_n_ after irradiance increases, and a smaller transient dip in A_n_ after irradiance decreases.

Leaves protect themselves from absorbed irradiance that is in excess of the capacity of photochemistry using non-photochemical quenching (NPQ). This protection, however, may come at a price. Sustained high levels of NPQ after irradiance decreases may result in transient limitations of the quantum efficiency of photosystem II for electron transport (ϕ_PSII_). Model calculations indicate that slow relaxation of NPQ could decrease canopy photosynthesis by ~13–24%^22^. NPQ has been shown to limit A_n_ in genotypes with faster NPQ buildup after irradiance increases^23^ or slower NPQ relaxation after irradiance decreases^24^. Thus, genotypes with constitutively low NPQ may have increased dynamic photosynthesis rates, principally as a result of less limitation on A_n_ following a decrease in irradiance.

In many plants, stomata open when irradiance increases. Typically, stomatal opening is slow, transiently limiting A_n_ during the irradiance increase^25^. Genotypes with constitutively high g_s_ may not experience this limitation^26^, and may therefore be more productive in environments with a high proportion of fluctuating irradiance, provided that water is not limiting.

We used several genotypes, i.e. plants containing point mutations, transformants, T-DNA insertion lines (SALK lines^27^) and naturally occurring accessions of *A. thaliana*, to analyse how metabolic (Rubisco activation, sucrose synthesis, NPQ) and diffusional (g_s_) limitations affect dynamic photosynthesis. In addition to measuring their steady-state photosynthetic irradiance and CO_2_ responses, we exposed these genotypes to stepwise increases and decreases in irradiance, while measuring gas exchange and chlorophyll fluorescence. To investigate the effects of Rca regulatory properties or concentrations, we used the transformant *rwt43* (lacks the α-isoform of Rca and is therefore ADP-insensitive^19^) and the mutant *rca-2*, which is due to a leaky allele mutation (decreased Rca concentration^28^). To analyze the effect of SPS, we studied the T-DNA mutant line *spsa1* (80% reduction in maximum SPS activity^29^). The effect of low NPQ was investigated by using *npq4-1* (lacks PsbS, greatly diminishing NPQ^30^) and *npq1-2* (lacks zeaxanthin deepoxidase and therefore violaxanthin, diminishing NPQ^31^). Effects of high and low g_s_ were analyzed by using *aba2-1* (impaired abscisic acid (ABA) synthesis, leading to constitutively high g_s_^32^) and the natural accession C24 (low g_s_^33^), respectively. The accession Col-0 is the wildtype background to all mutants and transformants used in this study and acts as a control line. This study indicates that wildtype isoform composition and amount of Rca, as well as g_s_ limit dynamic photosynthesis in *A. thaliana*, while wildtype levels of SPS and NPQ do not.

## Results

### Steady-state responses to irradiance and CO_2_ confirm genotypic effects on Rubisco activation state, sugar metabolism and stomatal conductance

**Figure 1:**
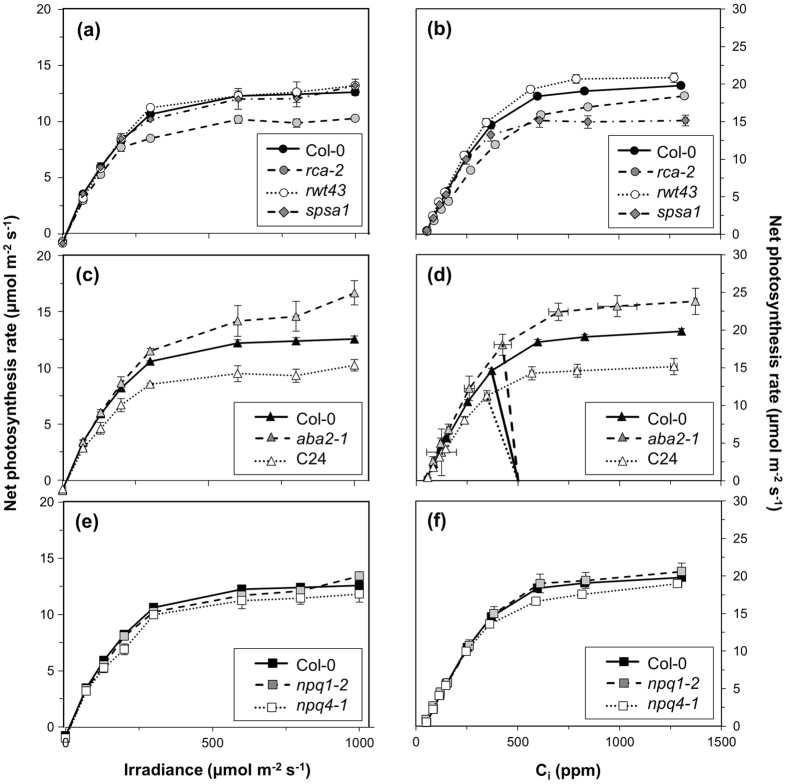
Irradiance and CO_2_ response of net photosynthesis rates in *rca-2*, *rwt43* and *spsa1* (**a,b**), *aba2-1* and C24 (**c,d**) and *npq1-2* and *npq4-1* (**e,f**). Col-0 is included in each panel for ease of comparison. In (**d**), supply lines^38^ between C_a_ = 500 and the corresponding C_i_ response curve of A_n_ are shown to emphasize stomatal effects of *aba2-1*, C24 and Col-0 on C_i_. Averages ± SEM, n = 5–15.

To characterize the steady-state behaviour of the different *A. thaliana* genotypes we measured their responses to irradiance and leaf internal CO_2_ concentration (C_i_). Rates of A_n_ in Col-0 were comparable to studies using plants grown under similar conditions^34^,^35^,^36^,^37^. In the mutant containing less Rca, *rca-2*, irradiance-saturated A_n_ was lower than for Col-0, and saturation occurred around 600 μmol m^−2^ s^−1^ (Fig. 1a). The lower C_i_ response on A_n_ in *rca-2* (Fig. 1b) resulted in significantly decreased maximum carboxylation rate by Rubisco (V_cmax_; −23%), maximum rate of electron transport (J_max_; −14%) and maximum rate of triose phosphate utilisation (TPU; −7%) compared to Col-0 (Table 1). Assimilation in the transformant lacking the α-isoform of Rca, *rwt43*, exhibited similar irradiance and C_i_ responses as in Col-0 (Fig. 1). In the mutant with less SPS (*spsa1*), A_n_ did not differ from Col-0 in its irradiance response (Fig. 1a), but was strongly reduced at high C_i_ (Fig. 1b), resulting in decreased J_max_ (−14%) and TPU (−23%). The ABA-deficient mutant, *aba2-1*, showed larger irradiance- and CO_2_-saturated photosynthesis rates compared to Col-0, while the accession C24 showed the opposite (Fig. 1c,d). Some parameters derived from C_i_ response curves were therefore larger in *aba2-1* (J_max_: +18%, TPU: +19%), while they were smaller in C24 (V_cmax_: −17%, J_max_: −20%, TPU: −22%). The supply lines^38^ (Fig. 1d) emphasize differences in g_s_ between C24, Col-0 and *aba2-1*: the steeper the slope, the smaller the difference between external CO_2_ concentration (C_a_) and C_i_, and the larger g_s_. Irradiance and C_i_ responses of photosynthesis of low-NPQ mutants (*npq1-2*, *npq4-1*) were similar to Col-0 (Fig. 1e,f), except for lower J_max_ in *npq4-1* (−7%). The response of quantum yield of photosystem II (ϕ_PSII_) to C_i_ largely paralleled that of A_n_, with the exception that ϕ_PSII_ decreased at high C_i_ in many genotypes (except *rca-2* and *npq4-1*; see Supplementary Fig. 1). This decrease in ϕ_PSII_ was most marked, and started at a lower C_i_, in *spsa1* (Supplementary Fig. 1a).

### Larger Rubisco activation state and g_s_ accelerate photosynthetic induction, while lower NPQ does not

**Figure 2:**
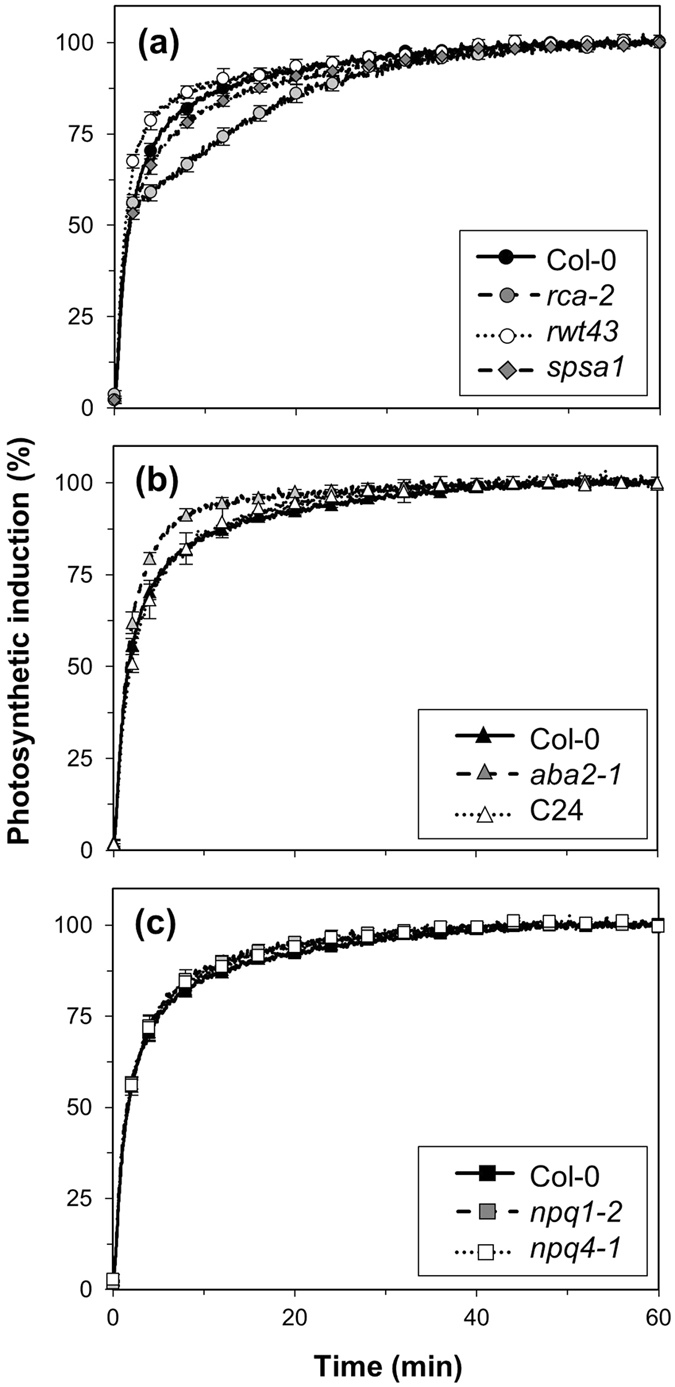
Photosynthetic induction after a step increase in irradiance from 0 to 1000 μmol m^−2^ s^−1^ in *rca-2, rwt43* and *spsa1* (**a**), *aba2-1* and C24 (**b**) and *npq1-2* and *npq4-1* (**c**). Col-0 is included in each panel for ease of comparison. Averages ± SEM, n = 5–15.

Next, we characterised the dynamic behaviour of leaf gas exchange by inducing photosynthesis in dark-adapted leaves using a stepwise increase to saturating irradiance (1000 μmol m^−2^ s^−1^). Rates of photosynthetic induction were initially similar between all genotypes (except *rwt43*) until ~60% induction was reached (Fig. 2). *rwt43* reached 50% of photosynthetic induction (t_A50_) significantly faster than Col-0 (Table 2). Induction remained faster in *rwt43* until it reached ~80% (Fig. 2a). In *rca-2*, the rate of induction slowed after 60% completion and then increased in a nearly linear fashion rather than the more exponential increase shown by all other genotypes (Fig. 2a). This increased the time to reach 90% of photosynthetic induction (t_A90_) by ~10 minutes compared to Col-0. *spsa1* showed slightly slower induction rates (Fig. 2a), increasing t_A90_ by ~5 min compared to Col-0. *aba2-1* exhibited faster induction, halving the t_A90_ of Col-0, while induction in C24 was identical to that of Col-0 (Fig. 2b). Induction in *npq1-2* and *npq4-1* was identical to Col-0 (Fig. 2c).

**Figure 3:**
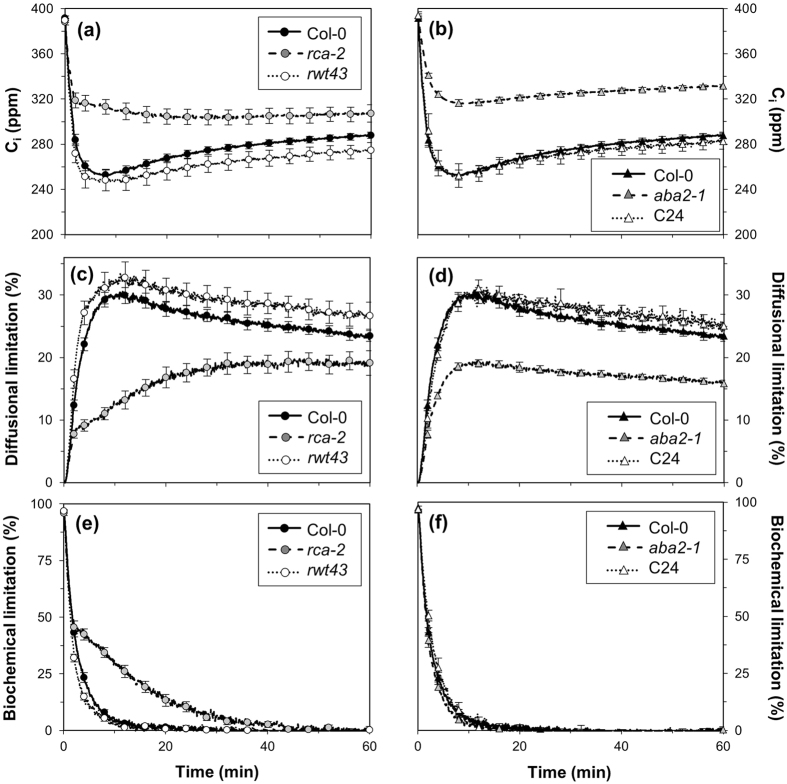
Leaf internal CO_2_ concentration (C_i_), diffusional limitation and biochemical limitation after a step increase in irradiance from 0 to 1000 μmol m^−2^ s^−1^ in Col-0, *rca-2* and *rwt43* (**a**,**c**,**e**) and Col-0, *aba2-1* and C24 (**b**,**d**,**f**). Averages ± SEM, n = 5–15.

To explain the differences between genotypes affecting Rubisco activation and g_s_, we looked at the time courses of C_i_, diffusional limitation and biochemical limitation. While C_i_ in Col-0 and *rwt43* dropped by ~130 ppm within 10 minutes and then increased by 30–40 ppm following stomatal opening, in *rca-2* it never dropped below its final value (Fig. 3a). Diffusional limitation reached its maximum within ~10 minutes in Col-0 and *rwt43* and then relaxed, while in *rca-2* its increase was much slower and levelled off after ~30 minutes (Fig. 3c). Biochemical limitation during induction relaxed almost completely within ~10 minutes in Col-0 and *rwt43*, while in *rca-2* it was generally greater and the same extent of relaxation took ~40 minutes (Fig. 3e). Comparing Col-0 and C24, the responses of C_i_ were almost indistinguishable, while in *aba2-1* the initial decrease in C_i_ was smaller, ranging from 50–60% of that found in Col-0 (Fig. 3b). Buildup and relaxation of diffusional limitation were much smaller in *aba2-1* (Fig. 3d), while relaxation of biochemical limitation was similar between Col-0, *aba2-1* and C24 (Fig. 3f).

Next to the dark-light transition discussed above, we also exposed leaves that had been adapted to low irradiance (hereafter: background irradiance) to stepwise increases in irradiance, namely 70 → 800 and 130 → 600 μmol m^−2^ s^−1^. The responses of A_n_ to these increases were qualitatively similar to those seen after the dark-light transition (Supplementary Fig. 2). *rwt43* exhibited a faster increase, and *rca-2* a much slower increase than Col-0 (Supplementary Fig. 2a,b). This reduced t_A50_, but not t_A90_, in *rwt43*, while t_A50_ and t_A90_ in *rca-2* were larger than Col-0 (Table 2). C24 tended to increase photosynthesis more slowly compared to Col-0 (Supplementary Fig. 2c,d), leading to a larger t_A50_ after the 70 → 800 μmol m^−2^ s^−1^ step increase and larger t_A50_ and t_A90_ after the 130 → 600 μmol m^−2^ s^−1^ step increase. Assimilation responses in NPQ and SPS mutants to those intermediate irradiance increases were similar to Col-0.

**Figure 4:**
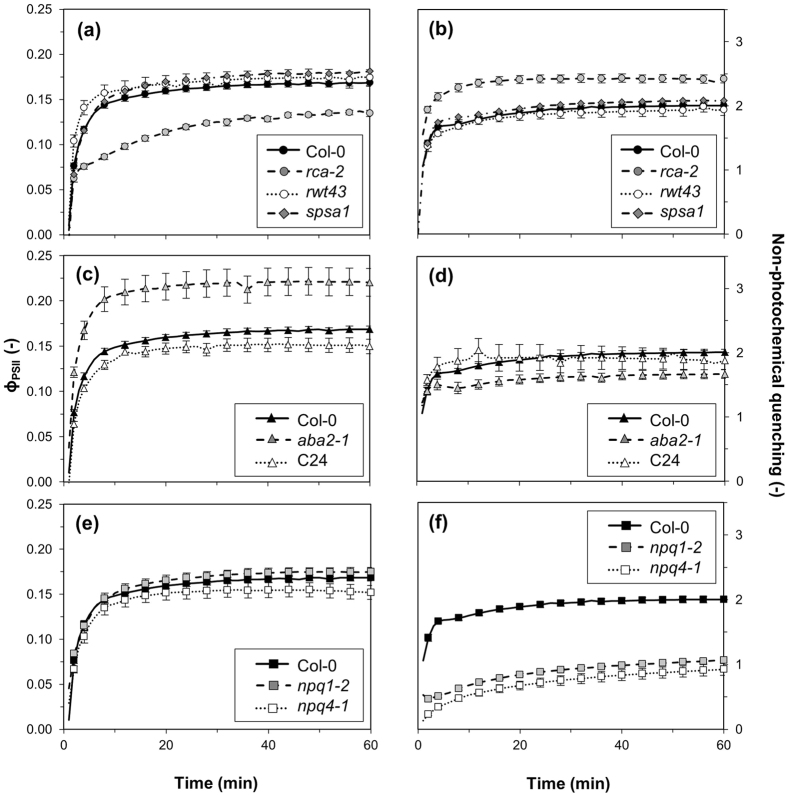
Quantum yield of photosystem II (ϕ_PSII_) and non-photochemical quenching (NPQ) after a step increase in irradiance from 0 to 1000 μmol m^−2^ s^−1^ in *rca-2, rwt43* and *spsa1* (**a**,**b**), *aba2-1* and C24 (**c**,**d**) and *npq1-2* and *npq4-1* (**e**,**f**). Col-0 is included in each panel for ease of comparison. Averages ± SEM, n = 5–15.

Apart from gas exchange dynamics, we also characterised changes in electron transport parameters after the stepwise 0–1000 μmol m^−2^ s^−1^ transition. Changes in ϕ_PSII_ largely paralleled those of A_n_ (Fig. 4). In *rwt43*, the increase in ϕ_PSII_ was slightly faster than in Col-0, while in *rca-2*, it was slower and steady-state ϕ_PSII_ was lower (Fig. 4a), paralleling its lower steady-state A_n_ (Fig 1a). Despite slightly larger ϕ_PSII_ throughout induction in *spsa1*, final values were not significantly different from Col-0 (P = 0.09, Fig. 4a). *aba2-1* showed increased steady-state ϕ_PSII_ levels, while in C24 they were reduced compared to Col-0 (Fig. 4c), similar to the differences in steady-state assimilation (Fig. 1c). In *npq4-1*, ϕ_PSII_ was slightly lower during induction than in *npq1-2* and Col-0 (*npq1-2* had similar ϕ_PSII_ trends and values during induction as Col-0; Fig. 4e). NPQ in *rca-2* increased more quickly to its steady-state level, which was larger than that of Col-0, *spsa1* and *rwt43* (Fig. 4b). NPQ in *aba2-1* was lower than in Col-0 and C24 (which were not significantly different from each other, Fig. 4d). As expected, *npq1-2* and *npq4-1* developed much lower NPQ levels than Col-0, and NPQ buildup was slower compared to Col-0, but similar in both *npq1-2* and *npq4-1* (Fig. 4f). Dark-adapted F_v_/F_m_ was 0.805 ± 0.002 (Avg ± standard error of the mean, SEM) in Col-0. In *rca-2*, C24 and *npq4-1*, F_v_/F_m_ was marginally, but significantly, smaller, possibly due to photoinhibition that was not completely removed by dark adaptation. In *spsa1*, it was slightly but significantly higher than in Col-0 (Supplementary Fig. 3).

### Isoform, amount and initial activation state of Rca affect the rate of Rubisco activation

**Figure 5:**
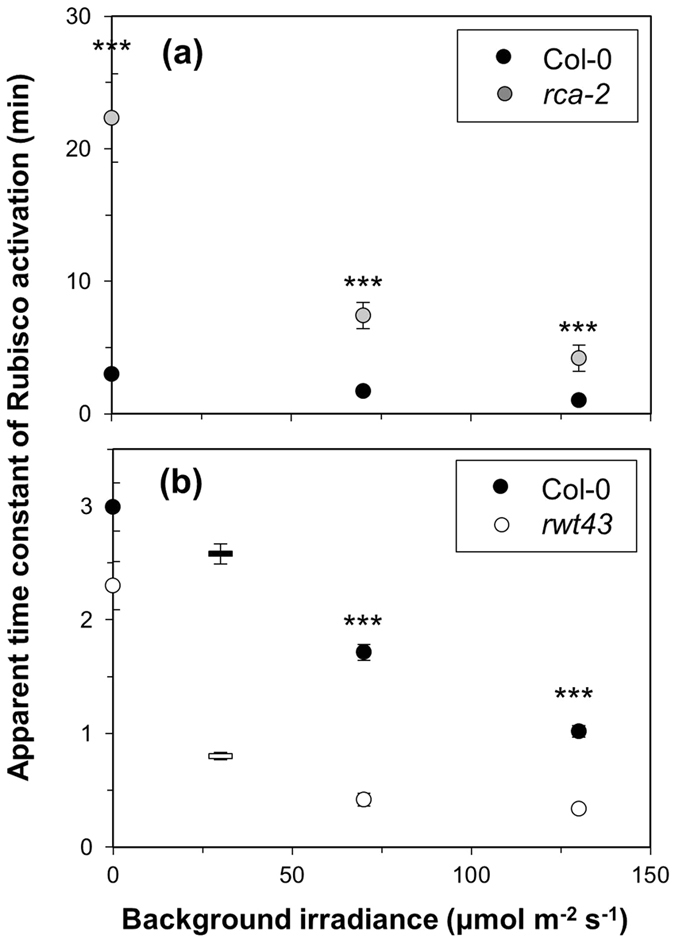
Apparent time constant of Rubisco activation in *rca-2* (**a**) and *rwt43* (**b**), compared to Col-0. Note the different scales of Y-axes in (**a**,**b**). Averages ± SEM, n = 5–15. Bars in (**b**) at 30 μmol m^−2^ s^−1^ background irradiance included from Carmo-Silva and Salvucci^42^. Stars denote significance levels of single genotypes compared to Col-0: ***P < 0.001.

The apparent time constants of Rubisco activation (*τ*_*R*_, the time to reach 63% of total change in Rubisco activation state), decreased with increasing background irradiance (Fig. 5). Genotypes differing in g_s_, NPQ and SPS did not differ from Col-0 in *τ*_*R*_. However, *τ*_*R*_ tended to be 17–28% larger in *spsa1* than in Col-0; *P*-values ranged from 0.07 to 0.09. Of the genotypes affecting Rca regulation, *rca-2* exhibited the biggest differences in *τ*_*R*_, both compared with Col-0 (P < 0.001 in all cases) and between background irradiances, with a *τ*_*R*_ of ~22 minutes in dark-adapted leaves decreasing to ~4 minutes in leaves adapted to an irradiance of 130 μmol m^−2^ s^−1^ (Fig. 5a). In *rwt43*, *τ*_*R*_ of dark-adapted leaves (2.3 min) was not significantly different to that of Col-0 (3.0 min; P = 0.08), but was significantly (P < 0.001) smaller at 70 and 130 μmol m^−2^s^−1^ background irradiance (Fig. 5b).

### Increases in initial g_s_ up to a threshold value accelerate photosynthetic induction

**Figure 6:**
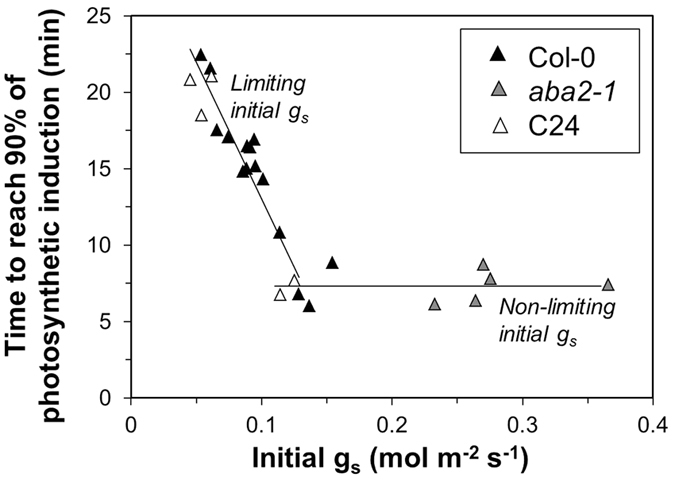
Relationship between initial g_s_ and the time to reach 90% of final photosynthesis rates after a step increase in irradiance (0–1000 μmol m^−2^ s^−1^) in single replicates of Col-0, *aba2-1* and C24.

Before and after stepwise increases in irradiance, g_s_ was considerably higher in *aba2-1* than in Col-0 and C24 (Supplementary Fig. 4). In dark-adapted leaves of Col-0 and C24, g_s_ was similar, but in leaves adapted to 70 or 130 μmol m^−2^ s^−1^, it was almost twice as high in Col-0 compared to C24. This spread in g_s_ was used to explore the threshold between a limiting and a non-limiting initial g_s_ for the subsequent rates of A_n_ increase. For example, after the 0 → 1000 μmol m^−2^ s^−1^ increase, t_A90_ was lower in plants with initially higher g_s_ up to ~0.13 mol m^−2^ s^−1^, but above 0.13 mol m^−2^ s^−1^ there was no further decrease in t_A90_ (Fig. 6). This shows that an initial g_s_ > 0.13 mol m^−2^ s^−1^ was non-limiting in this case. We also looked at various time points (t_A10_, t_A20_, etc.) after different low-to-high irradiance transitions (i.e. 0 → 1000, 70 → 800 and 130 → 600 μmol m^−2^ s^−1^) and found that the threshold between limiting and non-limiting initial g_s_ was between 0.09 and 0.17 mol m^−2^ s^−1^, with no discernible trend between time points or background irradiance levels.

Apart from the effect of initial g_s_ on the rate of A_n_ increase, we also analysed the effects of g_s_ increase after stepwise increases in irradiance (Supplementary Fig. 4). In C24 and Col-0, the increase in g_s_ after the 0 → 1000 μmol m^−2^ s^−1^ increase (until 60 minutes after the start of illumination) and t_A90_ correlated positively (Supplementary Fig. 5). Because initial g_s_ in *aba2-1* was high, it was non-limiting to rates of increase in photosynthesis after irradiance increases, and stomatal opening did not correlate with t_A90_ (data not shown).

### Lower NPQ and SPS do not increase transient photosynthesis after a decrease in irradiance

After step decreases in irradiance (600 → 200, 800 → 130 μmol m^−2^ s^−1^), relative changes in A_n_ were similar for all genotypes (Supplementary Fig. 6), and there were no significant differences in either post-illumination CO_2_ fixation or the post-illumination CO_2_ burst, including the NPQ mutants and *spsa1* (Supplementary Fig. 7).

## Discussion

Making use of the genetic diversity available for *A. thaliana*, we explored several possible physiological limitations of dynamic photosynthesis. This analysis revealed that altered Rubisco activation kinetics or stomatal conductance affect photosynthesis in a dynamic irradiance environment greatly, while alterations in non-photochemical quenching or sucrose synthesis do not.

Changes affecting Rca concentration (*rca-2*) or regulation (*rwt43*) had strong effects on dynamic photosynthesis. The observed effects were likely caused by different kinetics of Rubisco activation, as the initial increase in assimilation after dark-light transitions (first minute in Fig. 2a) was similar between genotypes, implying a similar limitation due to activation of RuBP regeneration (Sassenrath-Cole and Pearcy^7^ provided biochemical evidence for this). Furthermore, these genotypes had similar g_s_ (Supplementary Fig. 8). Lower steady-state irradiance and CO_2_ responses in *rca-2* may have been caused by a reduced steady-state activation of Rubisco^39^. Based on the dependency between maximum Rubisco activation state and Rca concentration reported by Mott and Woodrow^40^ and our estimation of V_cmax_ for *rca-2* (Table 1), we estimate that *rca-2* contains ~22% of wildtype Rca levels (Supplementary text 1). The effects on the rate of Rubisco activation of such low Rca content are apparent. In antisense or overexpressors of Rca in rice, a positive linear relationship between Rca concentration and the rate of photosynthetic induction was shown for various temperatures^41^, demonstrating the role of Rca concentration in controlling dynamic photosynthesis. Intriguingly, in our study *τ*_*R*_ decreased with background irradiance (Fig. 5). While this decrease was linear in Col-0, it resembled a negative exponential in *rwt43*. This is in agreement with data of Carmo-Silva and Salvucci^42^ (Fig. 5b). Previous studies have shown that Rubisco activation in Col-0 increased linearly with irradiance^42^,^43^,^44^, while in *rwt43*, Rubisco activation state did not change with increasing irradiance^42^; it was similar to Col-0 in dark-adapted leaves, but close to full activation in low irradiance^19^,^42^,^44^. Most likely differences in the activation state of Rca, rather than that of Rubisco, caused *τ*_*R*_ to decrease with background irradiance. Rca activity increased linearly between 0 and 300 μmol m^−2^ s^−1^ in intact spinach leaves^45^, and should be high in *rwt43* except in darkness (see above).

Compared to natural fluctuations in irradiance, stomata open and close slowly^46^. Low initial g_s_ can become a limitation to carbon fixation after a step change in irradiance^2^, because of comparably rapid activation of RuBP regeneration and Rubisco. The peak of this limitation is typically reached within ~10 minutes due to Rubisco activation without similarly large increases in g_s_, after which it relaxes due to stomatal opening (Fig. 3d). We note that the index of diffusional limitation should be refined with respect to changes in Rubisco activation during photosynthetic induction, as well as possible changes in mesophyll conductance (g_m_) during transients. With respect to g_m_, contrasting responses to irradiance have been reported (cf. refs 47 and 48); we therefore refrain from speculations on how it may have changed in our measurements but note that it may have affected the index of diffusional limitation. Nevertheless, we believe that diffusional limitation provides a useful qualitative tool to analyse the differences between the genotypes affecting Rubisco activation kinetics and g_s_.

The mutant with high initial g_s_ (*aba2-1*) did not show such large differences in stomatal opening (i.e. difference between initial and final g_s_; Supplementary Fig. 4), but still had much higher rates of A_n_ increases when irradiance was raised. Therefore, we argue that increasing the initial g_s_ is a simpler route to increasing dynamic photosynthesis than is increasing the rate of stomatal opening. Stomatal closure in low irradiance is an adaptive response to changing water supply and logical under non-irrigated field conditions, however for crops in well-watered situations, increasing g_s_ at the expense of water use may be a reasonable target to increase rates of dynamic photosynthesis. Also, the threshold between limiting and non-limiting g_s_ for rates of photosynthesis increase could be used as a phenotypic marker for breeding of cultivars with non-limiting g_s_ in fluctuating irradiance. In our analysis, this threshold proved to be consistent, independent of the time point after stepwise increases in irradiance and level of background irradiance. Previous findings indicate that this threshold shows no diurnal variation^26^, and that it is unchanged by water stress^26^ or growth light conditions^49^. An open question that remains is whether the threshold is species-specific^26^ or not^49^. It is likely that a high initial g_s_ correlates with constitutively high g_s_ (i.e. stomata are more open and less sensitive to changes in irradiance), and faster responses of A_n_ to an increasing irradiance could be reached at the expense of lower intrinsic water use efficiency. Rapid screening for high g_s_ could be achieved by thermal imaging^50^.

In Col-0, rates of NPQ buildup after a dark-light transition were similar to those seen in previous studies^51^,^52^, while mutants *npq1-2* (lacking violaxanthin de-epoxidase^31^) and *npq4-1* (lacking PsbS^30^) exhibited a much lower buildup of NPQ. However, they showed negligible differences in gas exchange to Col-0, neither in their steady-state responses to irradiance and CO_2_ (Fig. 1e,f) nor in their responses to step increases in irradiance (Fig. 2c, Supplementary Fig. 2e,f). Similar to our findings, reduced PsbS content in transgenic rice plants strongly reduced NPQ but had limited effects on carbon gain during a 5-min induction period^23^. In contrast, overexpressors with 2–4 fold increases in PsbS showed ~15% lower A_n_ during induction, demonstrating that increased energy dissipation can have adverse effects on assimilation^23^. Antisense mutants with reduced thylakoid membrane K^+^ flux capacities showed less rapid relaxation of NPQ after irradiance decreases, reducing electron transport and assimilation^24^. Our data revealed no differences between *npq1-2*, *npq4-1* and Col-0 with respect to post-illumination CO_2_ fixation (Supplementary Fig. 7), and therefore show that unlike the rate of NPQ relaxation^22^,^24^, an initially low level of NPQ does not increase carbon gain directly after decreases in irradiance.

Irradiance-dependent activation of SPS is genotype-specific, and *A. thaliana* belongs to a group of species with low light/dark modulation of the enzyme^53^. This suggests that in the wildtype, SPS activity does not limit photosynthetic induction–however, in a plant with strongly reduced SPS concentration it might. We tested this possibility in the T-DNA mutant *spsa1*, which has a 80% lower maximum SPS activity than Col-0^29^. Similar to our findings, Sun *et al*.^29^ found no photosynthetic differences between *spsa1* and Col-0, except for a strong reduction in CO_2_-saturated A_n_ (−23%). Importantly, the decrease in SPS hardly affected photosynthetic responses to fluctuating irradiance. The only significant difference was a longer time to reach 90% of full induction after dark-light transitions (Table 2). However, no such differences were observed in transitions from low to higher irradiance. *spsa1* would probably show decreased rates of dynamic photosynthesis in elevated CO_2_ concentrations. Furthermore, it may be that the absence of a measurable effect of *spsa1* on the post-illumination CO_2_ burst, which is partly affected by the rate of sucrose synthesis^21^, was masked by the photorespiratory portion of the CO_2_ burst, which is most pronounced in C_3_ plants^5^. Also, reduced levels of SPS in species that exhibit strong light/dark modulation of SPS (e.g. barley, maize, spinach and sugarbeet^53^) would probably have a stronger negative effect on photosynthetic induction than shown here for *A. thaliana*.

The relationship between ϕ_PSII_ and C_i_ in C_3_ photosynthesis contains three phases: When A_n_ is (a) limited by Rubisco, ϕ_PSII_ increases with C_i_; when A_n_ is (b) limited by RuBP regeneration, ϕ_PSII_ is constant with increases in C_i_ and when A_n_ is (c) limited by TPU, ϕ_PSII_ decreases with increasing C_i_^54^,^55^. Most genotypes in our study did not show the plateau in ϕ_PSII_ that would signify a phase of RuBP regeneration limitation, with *spsa1* showing an extreme form of that behaviour (Supplementary Fig. 1). This suggests that (a) TPU occurs at a lower C_i_ than visible from gas exchange, (b) different limitations occur simultaneously within different layers of the leaf, (c) changes in the rate of cyclic electron transport around photosystem I and/or strength of alternative electron sinks or (d) with increasing C_i_ during the phase of limitation by RuBP regeneration photosynthetic electron transport is sometimes restricted, and ϕ_PSII_ is reduced, due to the increased inhibition of starch synthesis following the inhibition of phosphoglucoisomerase by phosphoglycerate^56^. However, these results have to be interpreted with caution because the number of data points between the end of Rubisco limitation and the onset of TPU was limited and more data may lead to different conclusions.

In conclusion, in *A. thaliana*, the presence of the redox-regulated α-isoform of Rca in the wildtype, and wildtype levels of g_s_, are limiting for dynamic photosynthesis. Furthermore, reductions in Rca strongly decrease (dynamic) photosynthesis. We also show that wildtype levels of NPQ and SPS are not limiting in *A. thaliana*. This suggests Rca and g_s_ as targets for improvement of photosynthesis in fluctuating irradiance.

## Methods

### Plant material

Seeds of *npq4-1*, *spsa1* (SALK_148643C) and *rca-2* (SALK_003204C) were obtained from NASC (University of Nottingham, Loughborough, UK^57^). C24 (CS76106) was obtained from the Arabidopsis Biological Resource Center (ABRC, Ohio State University, USA). Seeds of Col-0 and *aba2-1* were obtained from Corrie Hanhart (Wageningen University, the Netherlands), *npq1-2* was obtained from Dr. Shizue Matsubara (Forschungszentrum Jülich, Germany) and *rwt43* was obtained from Dr. Elizabete Carmo-Silva (Rothamsted Research, UK).

### Growth conditions

Plants were grown in 0.37 L pots using soil with a 4:1 peat:perlite mixture. Pots were placed on irrigation mats, and mats were saturated daily to full capacity. Plants were fertilized weekly using a nutrient solution especially developed for Arabidopsis^58^. To inhibit algal growth, the soil was covered with black plastic film. Plants were grown in a growth chamber in short-day conditions (8 hours of light) to delay flowering^59^ and thus ensure that leaves were large enough for gas-exchange measurements. Irradiance was 172 ± 4 μmol m^−2^ s^−1^ as supplied by LED lights (GreenPower LED production module deep red/white 120; Philips, Eindhoven, the Netherlands; Supplementary Fig. 9). Temperature was 23/18 °C (day/night) and relative humidity was 70%. Mutants lacking ABA (*aba2-1*) were sprayed with an aqueous solution containing 10 μmol mol^−1^ ABA (Sigma, St. Louis, U.S.A.) when plants were 2, 4 and 6 weeks old. This increases rosette growth compared to untreated *aba2-1* plants (data not shown). There was a period of 15 days between the last application of ABA and the first measurements on *aba2-1* plants.

Single genotypes were grown sequentially (approx. one batch per week). Five plants per batch were used for measurements. To monitor the quality of the growth system over time, Col-0 was grown in three batches, each batch separated by several weeks. The number of replicates was therefore 15 for Col-0, and 5 for all other genotypes. The growth system produced very reproducible photosynthetic phenotypes of Col-0 (Supplementary Fig. 10).

### Measurements

Measurements were performed using the LI-6400 portable photosynthesis system (Li-Cor Biosciences, Lincoln, Nebraska, USA) equipped with the leaf chamber fluorometer (Part No. 6400-40) on single leaves of plants that were 6–8 weeks old. Leaves large enough to cover the leaf chamber gasket (area: 2 cm^2^, diameter: 1.6 cm) were used. Conditions in the cuvette were as follows: 23 °C air temperature, 70% relative humidity, 90/10% red/blue light mixture and 500 μmol s^−1^ air flow rate. The choice of flow rate was a compromise between getting a fast time response of the measuring system (necessary in dynamic gas exchange studies), and the difference in CO_2_ concentration between sample and reference air stream. Except for the CO_2_-response curves, the external CO_2_ mole fraction was kept at 400 ppm. The oxygen mole fraction was always 21%.

### Stepwise increases in irradiance

Leaves were adapted to several background irradiances (0, 70 or 130 μmol m^−2^ s^−1^) for 30–60 minutes (until A_n_ and g_s_ had visibly reached a steady state), and then exposed to single-step increases in irradiance, namely 0 → 1000, 70 → 800 and 130 → 600 μmol m^−2^ s^−1^. These intensities were chosen, after preliminary irradiance-response curves on Col-0 had shown that all but the highest (1000 μmol m^−2^ s^−1^) intensity were in the sub-saturating range (Supplementary Fig. 11). Gas exchange was logged nominally every second. Logging was stopped when g_s_ reached a new steady state (this was assessed visually, and took a minimum of 30 minutes after the step increase), or 60 minutes after switching to 1000 μmol m^−2^ s^−1^. Before and after the 0 → 1000 μmol m^−2^ s^−1^ increase, ϕ_PSII_ and NPQ were measured, using a measuring beam intensity of ~1 μmol m^−2^ s^−1^ and a saturating pulse of ~7600 μmol m^−2^ s^−1^ intensity and 1 s duration. In preliminary measurements on Col-0, the saturating pulse was sufficient to saturate F_m_’ in leaves adapted to 1000 μmol m^−2^ s^−1^ (assessed following the manufacturer’s recommendations for calibrating the saturating pulse: F_m_’ was not increased when using saturating pulses of intensity higher than 7600 μmol m^−2^ s^−1^). The F_o_ and F_m_ relative fluorescence yields were measured in dark-adapted leaves. After the increase in irradiance, the F_m_’ relative fluorescence yield was measured every minute for the first ten minutes, and every two minutes thereafter. The regular application of saturating flashes transiently increased the leaf temperature by 0.4–0.7 °C across genotypes (temperature traces of Col-0 are representative of all genotypes, Supplementary Fig. 12). Also, our data (Kaiser *et al*., unpublished) indicate that the regular application of saturating flashes of similar intensity and frequency in tomato (*Lycopersicon esculentum*) had no effects on leaf gas exchange during photosynthetic induction. The steady-state relative fluorescence yield, F_s_, was measured continuously. Dark-adapted F_v_/F_m_, ϕ_PSII_ and NPQ were calculated as F_v_/F_m_ = (F_m_ − F_o_)/F_m_, ϕ_PSII_ = (F_m_’ − F_s_)/F_m_’ and NPQ = (F_m_ − F_m_’)/F_m_’, respectively.

During transients, g_m_ and mitochondrial respiration (R_d_) were assumed to be constant because, to our knowledge, changes in g_m_ and R_d_ have never been assessed for irradiance transients. R_d_ in the light was considered similar to genotype-specific steady-state respiration in the dark; this assumption is supported by measurements on several species^60^. For g_m_, a value of 0.2 mol m^−2^ s^−1^ was assumed for all genotypes, which is an average of three values determined on Col-0 of comparable photosynthetic capacity^35^,^61^.

The time to reach 50 and 90% (i.e. t_50_ and t_90_) of steady-state A_n_ was calculated for each irradiance increase. To increase robustness of these indices to experimental noise and outliers, time series were smoothed using a local polynomial regression^62^ with a span of 5%. This means that, for each point in the time series, a polynomial of degree two was fitted using weighted least squares to a data window of size equal to 5% of the total size of the time series; the weight assigned to each point decreases with the distance from the central point.

### Calculation of diffusional limitation, biochemical limitation and the apparent time constant of Rubisco activation

To calculate several parameters, A_n_ was corrected for transient changes in chloroplast CO_2_ concentration (C_c_). For diffusional limitation, A_n_ was multiplied by the relative rate by which A_n_ would increase if C_c_ during induction was equal to ambient CO_2_ concentration, C_a_ (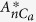):


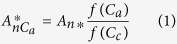


Where f(C_a_) is the steady-state value of A_n_ at C_a_ (i.e. at 400 ppm), and f(C_c_) is the steady-state value of A_n_ at C_c_. The relative effects of C_c_ on A_n_ were taken from steady-state A_n_/C_c_ response curves by fitting local polynomial regressions (LOESS) in the range 50–500 ppm (Supplementary Fig. 13). Diffusional limitation was then determined as:


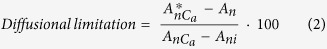


Where A_nCa_ is the steady-state value of A_n_ at C_a_ and A_ni_ is the initial steady-state rate of A_n_. Diffusional limitation is therefore a combination of possible limitations due to g_s_ and g_m_ during induction and in the steady state (i.e. it does not decrease to 0% at the end of the time course). For biochemical limitation and *τ*_*R*_, A_n_ was multiplied (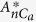) by the relative rate by which A_n_ would increase if transient C_c_ was equal to final, steady-state C_c_ (C_cf_), following Woodrow and Mott^15^:


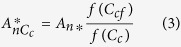


Where f(C_cf_) is the solution for A_n_ at C_cf_. Biochemical limitation was calculated after Allen and Pearcy^63^:


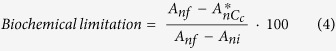


Throughout induction, biochemical limitation decreases from 100 to 0%, and therefore indicates the additional limitation imposed on A_n_ due to incomplete activation of several enzymes. Biochemical and diffusional limitations do not sum up to 100%, and are distinct. The apparent time constant of Rubisco activation (*τ*_*R*_) was calculated after Woodrow and Mott^15^:


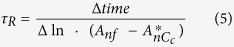


The range of timepoints (Δ*time*) for calculating *τ*_*R*_ differed between background irradiances (Supplementary Fig. 14), and in some cases between genotypes. This was due to differences in the rate of change of photosynthesis, and included 120 data points in the case of 0 → 1000 μmol m^−2^ s^−1^ (all genotypes) and 40 (for *rwt43*) or 60 (all other genotypes) in the case of 70 → 800 and 130 → 600 μmol m^−2^ s^−1^. These ranges were selected by visual inspection. The average root mean squared error of the linear fits was 1.2 μmol m^−2^ s^−1^ (range: 1.0–3.0 μmol m^−2^ s^−1^).

### Stepwise decreases in irradiance

Irradiance was decreased in the following steps: 800 → 130 and 600 → 200 μmol m^−2^ s^−1^. From the CO_2_ exchange data, post-illumination CO_2_ fixation^64^ and post-illumination CO_2_ bursts^65^ were quantified. The former implies that photosynthesis is above the final steady-state value during the transient, while the latter implies a lower assimilation rate than at steady state. Values were estimated by integrating the difference between time series of photosynthesis and the final steady-state value^66^.

### Irradiance response curves

When A_n_ was at a steady state, i.e. before step changes in irradiance or at the end of a measurement sequence, 120 data points were used to extract average A_n_ at a given irradiance. The resulting values were used to construct steady-state irradiance response curves.

### CO_2_ response curves

Leaves were adapted to 1000 μmol m^−2^ s^−1^ for ~30 min and 500 ppm C_a_. C_a_ was then decreased stepwise until 50 ppm, each step taking 2–3 minutes. Thereafter, C_a_ was raised to 500 ppm, and after waiting for ~15 minutes, leaves were exposed to stepwise increases in C_a_ until 1500 ppm, each step taking ~4 minutes. Values were logged every 5 s and the last 60 s of every CO_2_ step used to calculate average ± SEM of C_i_ and A_n_. ϕ_PSII_ was determined at the end of each step as described above. Photosynthesis in all genotypes was corrected for CO_2_ leaks using dried leaves of Col-0^54^. Parameters V_cmax,_ J_max_, and TPU were calculated after Sharkey *et al*.^55^.

### Statistical analysis

Each genotype was compared to Col-0 using a Student’s *t*-test (Microsoft Excel, function t.test, assuming 2-tailed distribution and two-sample equal variance).

## Additional Information

**How to cite this article**: Kaiser, E. *et al*. Metabolic and diffusional limitations of photosynthesis in fluctuating irradiance in *Arabidopsis thaliana*. *Sci. Rep*. **6**, 31252; doi: 10.1038/srep31252 (2016).

## Supplementary Material

Supplementary Information

## Figures and Tables

**Table 1:** Parameters derived from C_i_ response curves of A_n_.

	V_cmax_	J_max_	TPU	RMSE
Col-0	53 ± 1	100 ± 2	7.1 ± 0.1	0.93 ± 0.04
*rca-2*	40 ± 1***	86 ± 2***	6.7 ± 0.1 n.s.	0.95 ± 0.11 n.s.
*rwt43*	57 ± 3 n.s.	105 ± 5 n.s.	7.5 ± 0.2 n.s.	0.98 ± 0.07 n.s.
*spsa1*	54 ± 4 n.s.	86 ± 5**	5.5 ± 0.3***	0.85 ± 0.06 n.s.
*aba2-1*	58 ± 3 n.s.	118 ± 6***	8.5 ± 0.6**	1.12 ± 0.11 n.s.
C24	44 ± 2**	79 ± 4***	5.5 ± 0.4***	0.76 ± 0.07*
*npq1-2*	52 ± 3 n.s.	101 ± 5 n.s.	7.4 ± 0.4 n.s.	0.95 ± 0.08 n.s.
*npq4-1*	53 ± 1 n.s.	92 ± 2*	6.8 ± 0.2 n.s.	0.98 ± 0.03 n.s.

V_cmax_, maximum caboxylation rate by Rubisco (μmol CO_2_ m^−2^ s^−1^); J_max_, maximum rate of electron transport in the absence of regulation (μmol electrons m^−2^ s^−1^); TPU, maximum rate of triose phosphate utilisation (μmol CO_2_ m^−2^ s^−1^). The root mean squared error (RMSE, μmol CO_2_ m^−2^ s^−1^) of the differences between measurement and model during curve fitting^55^ is shown as an estimation of the overall goodness of fit. Averages ± SEM, n = 5–15. Stars within columns denote significance levels compared to Col-0: ***P < 0.0001, **P < 0.01, *P < 0.05. Absence of stars denotes lack of significant difference with Col-0 (P > 0.05).

**Table 2:** Time (minutes) to reach 50 and 90% of steady-state photosynthesis rates (t_A50_, t_A90_) after step increases in irradiance.

Genotype	0 → 1000 μmol m^−2^ s^−1^		70 → 800 μmol m^−2^ s^−1^		130 → 600 μmol m^−2^ s^−1^	
	t_A50_	t_A90_	t_A50_	t_A90_	t_A50_	t_A90_
Col-0	1.6 ± 0.1	14.7 ± 1.2	1.3 ± 0.1	10.2 ± 1.1	0.6 ± 0.0	9.0 ± 2.2
*rca-2*	1.5 ± 0.2	25.5 ± 1.5***	6.3 ± 0.4***	30.9 ± 2.0***	4.0 ± 0.7***	29.8 ± 1.7***
*rwt43*	1.2 ± 0.1**	14.2 ± 2.6	0.5 ± 0.0***	16.2 ± 6.1	0.3 ± 0.0***	18.8 ± 6.1
*spsa1*	1.6 ± 0.1	19.5 ± 1.3*	1.3 ± 0.1	14.1 ± 7.2	0.6 ± 0.1	13.7 ± 6.9
*aba2-1*	1.4 ± 0.1	7.3 ± 0.5**	1.3 ± 0.1	7.7 ± 2.6	0.8 ± 0.1	15.1 ± 5.8
C24	1.9 ± 0.1	15.0 ± 3.2	1.7 ± 0.3*	13.3 ± 2.7	0.9 ± 0.2*	29.4 ± 5.1***
*npq1-2*	1.4 ± 0.1	11.7 ± 1.7	1.3 ± 0.1	10.7 ± 2.9	0.7 ± 0.0	14.6 ± 8.6
*npq4-1*	1.5 ± 0.1	14.8 ± 2.6	1.1 ± 0.1	6.1 ± 0.7	0.6 ± 0.0	15.3 ± 11.0

Averages ± SEM, n = 5–15. Stars within columns denote significance levels compared to Col-0: ***P < 0.0001, **P < 0.01, *P < 0.05. Absence of stars denotes lack of significant difference with Col-0 (P > 0.05).
