# Supplementary material for: Metabolic and diffusional limitations of photosynthesis in fluctuating irradiance in Arabidopsis thaliana
Source: Sci Rep. 2016 Aug 9;6:31252. doi: 10.1038/srep31252 (PMC4977489; doi:10.1038/srep31252)
Supplement: Supplementary Information [file srep31252-s1.pdf]

Supplementary material

Metabolic and diffusional limitations of photosynthesis in fluctuating irradiance in *Arabidopsis thaliana*

Elias Kaiser, Alejandro Morales, Jeremy Harbinson, Ep Heuvelink, Aina E. Prinzenberg & Leo F.M. Marcelis

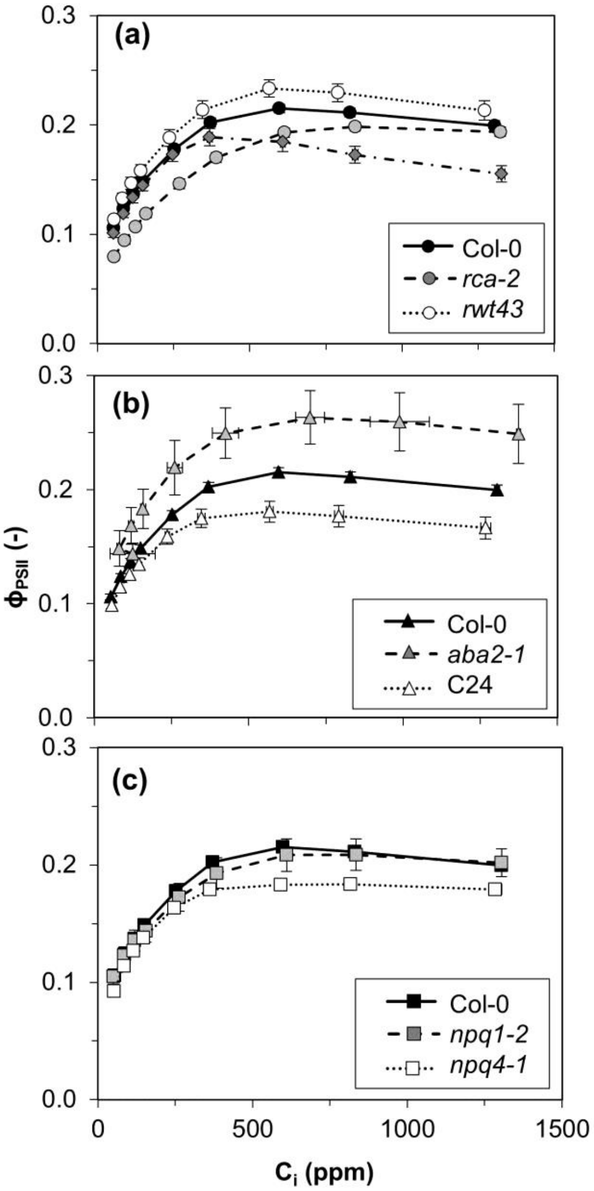

Supplementary Fig. 1. CO_2 response of ϕ_{PSII} in Arabidopsis genotypes. Averages ± SEM, n = 5-15

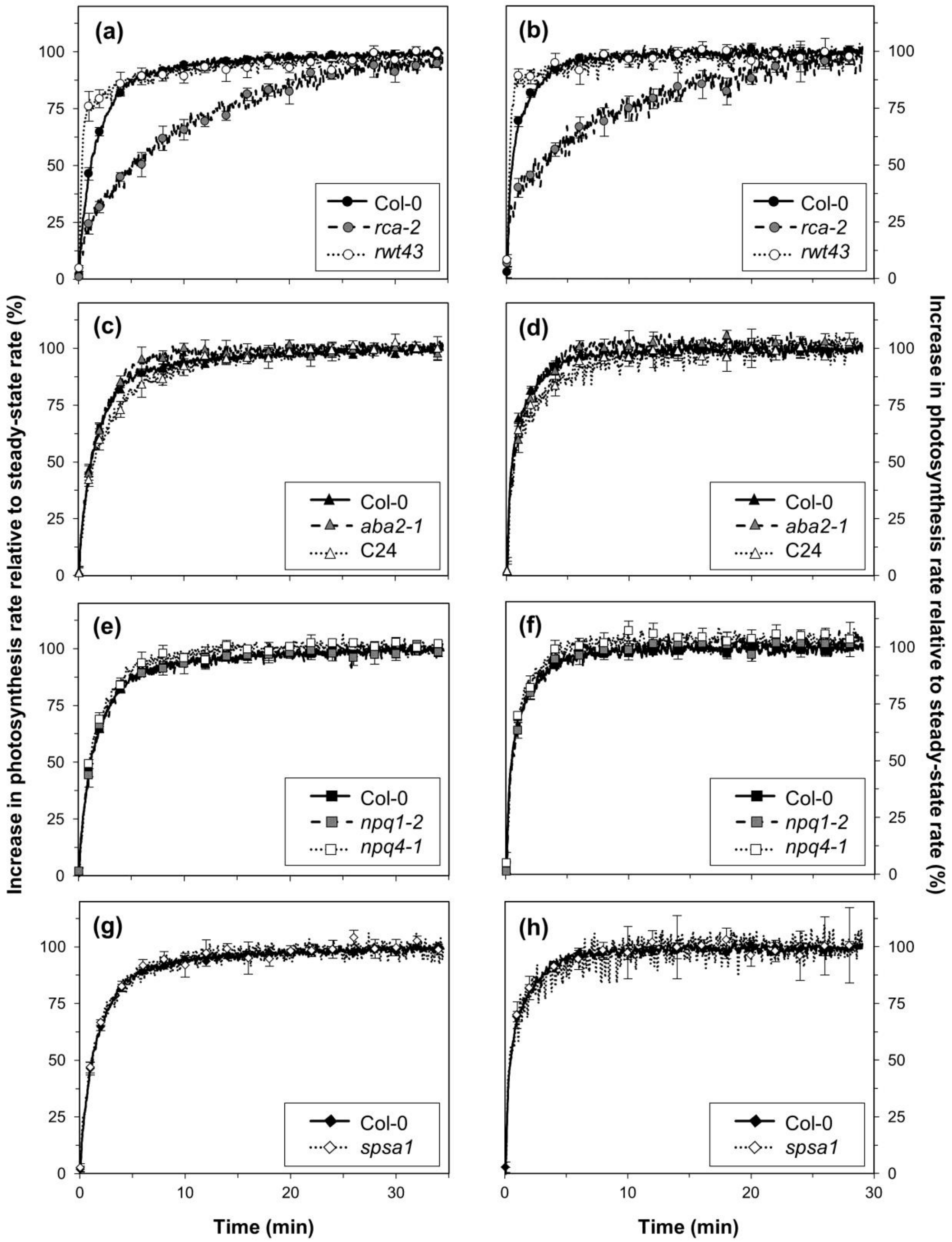

Supplementary Fig. 2. Relative responses of net photosynthesis rates to increases in irradiance, from 70 to 800 (left panel: a, c, e, g) and from 130 to 600 µmol m^{-2} s^{-1} (right panel: b, d, f, h). Averages ± SEM, n = 5-15

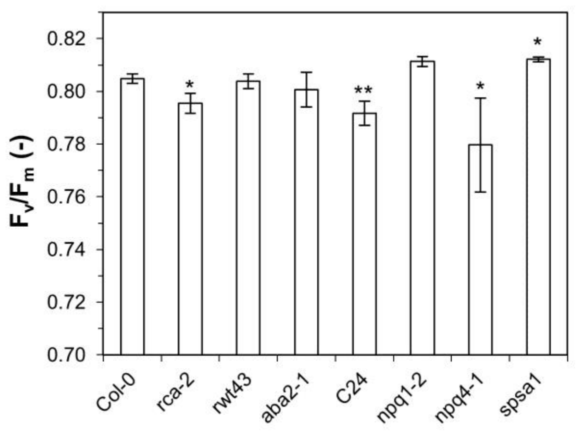

Supplementary Fig. 3. Dark-adapted F_v/F_m in Arabidopsis genotypes after 60 minutes of dark adaptation. Stars denote significant difference from Col-0, as P<0.05 (*) and P<0.01 (**). Averages ± SEM, n = 5-15

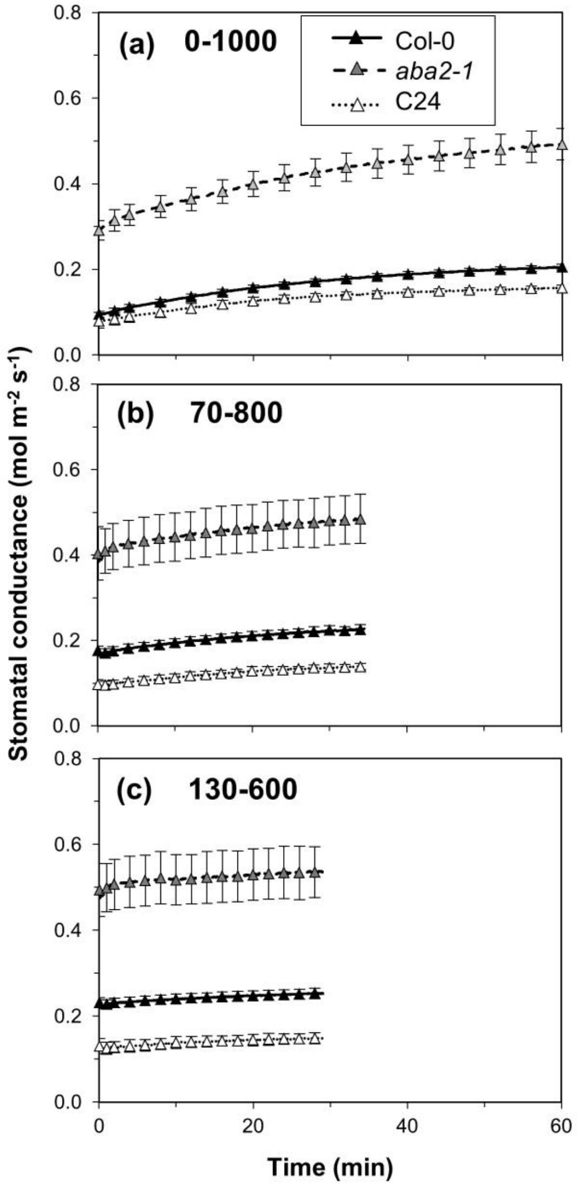

Supplementary Fig. 4. Increases in stomatal conductance in Col-0, *aba2-1* and C24 after step increases in irradiance, a) 0→1000, b) 70→800 and c) 130→600 µmol m^{-2} s^{-1}. Averages ± SEM, n = 5-15

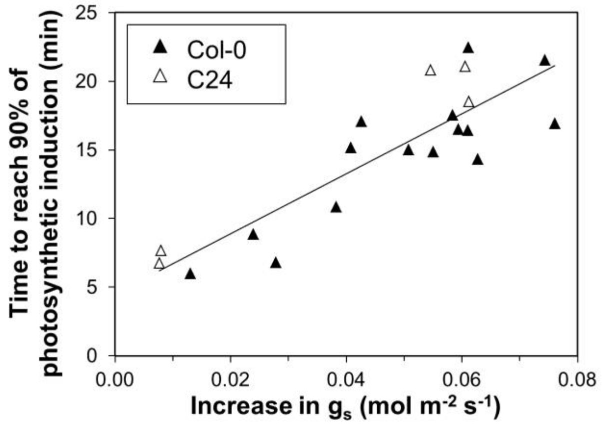

Supplementary Fig. 5. Relationship between the increase in g_s and the time to reach 90% of final photosynthesis rates after a step increase in irradiance (0-1000 μmol m^{-2} s^{-1}) in single replicates of Col-0 and C24 (R^2 = 0.75)

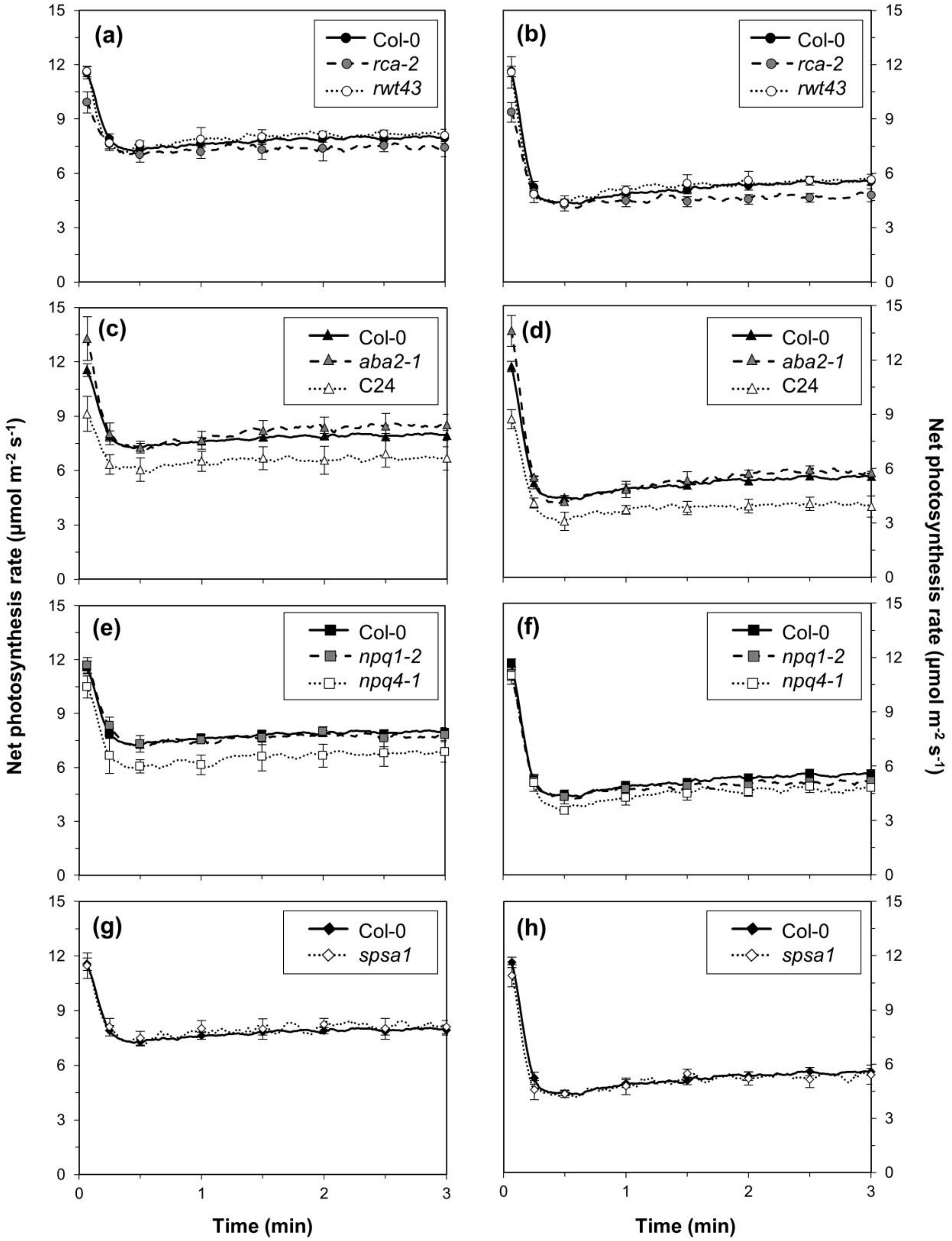

Supplementary Fig. 6. Responses of net photosynthesis rates to step decreases in irradiance, from 600 to 200 (left panel: a, c, e, g) and from 800 to 130 µmol m^{-2} s^{-1} (right panel: b, d, f, h). Averages ± SEM, n = 5-15

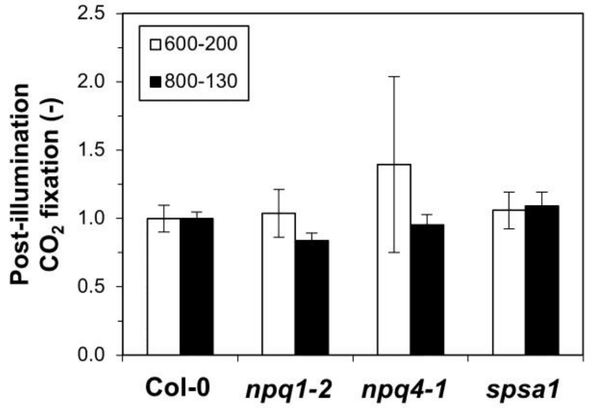

Supplementary Fig. 7. Relative post-illumination CO_2 fixation in Col-0, *npq1-2*, *npq4-1* and *spsa1*. Values are expressed relative to Col-0, which was 52 ± 5 μmol m^{-2} after 600→200 μmol m^{-2} s^{-1} step decreases (white bars) and 76 ± 3 μmol m^{-2} after 800→130 μmol m^{-2} s^{-1} step decreases (black bars). Averages ± SEM, n = 5-15

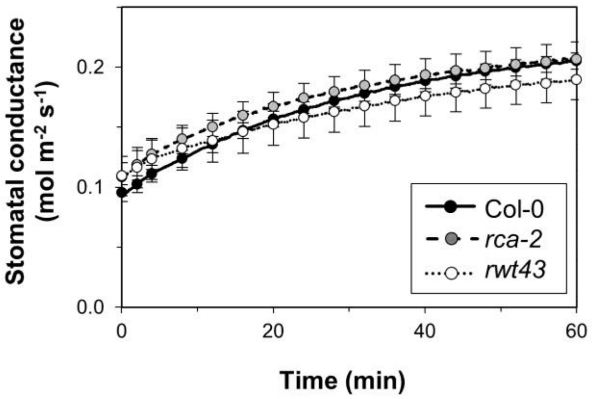

Supplementary Fig. 8. Increases in stomatal conductance in genotypes differing in Rubisco activation rate after a 0-1000 μmol m^{-2} s^{-1} step increase

Supplementary Text 1: Calculation of Rubisco activase content

Mott and Woodrow[1] calculated the maximum fraction of Rubisco activation (f_{RB}, achieved at ambient CO_2 concentration and saturating irradiance) from the amount of RCA (mg m^{-2}) in a non-linear manner as follows:

$$f_{RB} = \frac{RCA}{RCA + K_A^{RCA}}, \qquad \text{(S1)}$$

where K_A^{RCA} (mg m^{-2}) is the amount of Rubisco activase at which half of the maximum fraction of Rubisco activation is achieved. Carmo-Silva and Salvucci[2] reported, for leaves of *A. thaliana* Col-0, grown at similar conditions to the plants in our experiments, a maximum fraction of Rubisco activation of 0.91 (i.e. Rubisco is never fully activated). With the value of K_A^{RCA}= 12.3 mg m^{-2} reported by Mott and Woodrow[1] we can derive that the amount of Rubisco activase in Col-0 is 124.4 mg m^{-2}. Assuming that the total amount of Rubisco in Col-0 and *rca-2* is the same, we deduce the amount of Rubisco activase using Equation S1 and our values of V_{cmax} in Col-0 (53±1 µmol m^{-2} s^{-1}) and *rca-2* (40±1 µmol m^{-2} s^{-1}), which results in a value of 27 mg m^{-2}, that is, 22% of the wildtype level.

References

1 Mott, K. A. & Woodrow, I. E. Modelling the role of Rubisco activase in limiting non-steady-state photosynthesis. *Journal of Experimental Botany* **51**, 399-406 (2000).

2 Carmo-Silva, A. E. & Salvucci, M. E. The regulatory properties of Rubisco activase differ among species and affect photosynthetic induction during light transitions. *Plant Physiology* **161**, 1645-1655, doi:10.1104/pp.112.213348 (2013).

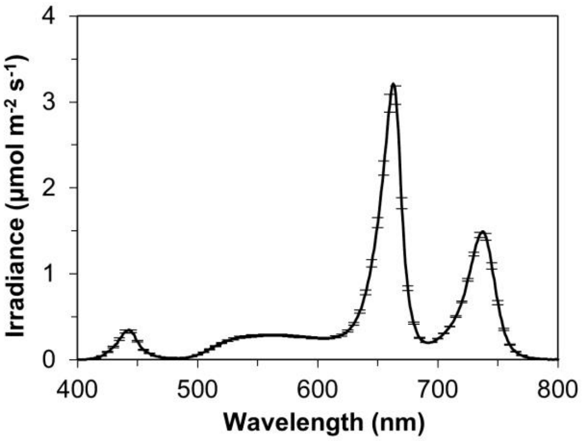

Supplementary Fig. 9. Irradiance spectrum in the growth chamber. Average ± SEM, n = 4

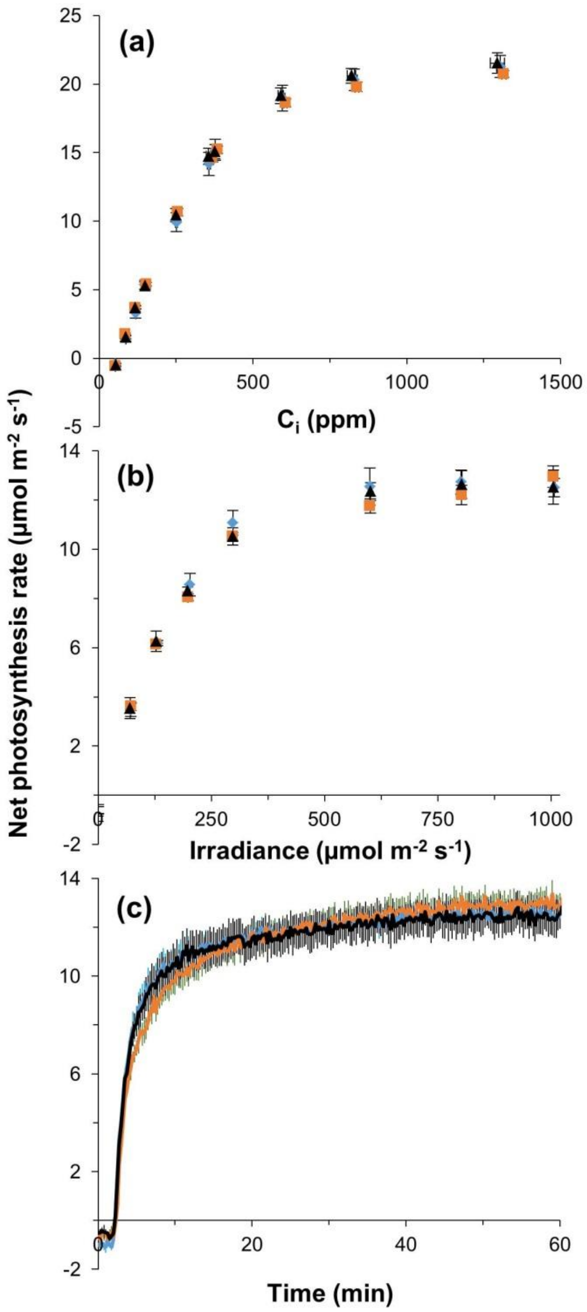

Supplementary Fig. 10. CO_2 response (a), irradiance response (b) and photosynthetic induction (c) in three batches of Col-0, grown sequentially in the same growth system. Batch 1, blue symbols; batch 2, orange symbols; batch 3, black symbols. Average ± SEM, n = 5

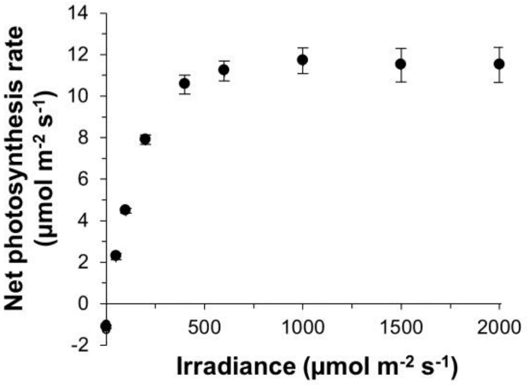

Supplementary Fig. 11. Irradiance response curves of net photosynthesis rate determined on leaves of Col-0 prior to starting the main experiment. These data were used to determine the irradiance levels to be used during for stepwise increases and decreases in irradiance

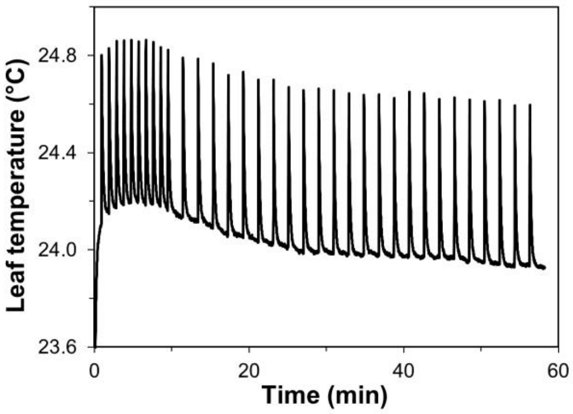

Supplementary Fig. 12. Time course of leaf temperature on a leaf of Col-0 after a stepwise increase from 0 to 1000 µmol m^{-2} s^{-1}. The spikes in leaf temperature are the results of the regular application of saturating flashes used to determine F_m', and are representative of all genotypes tested

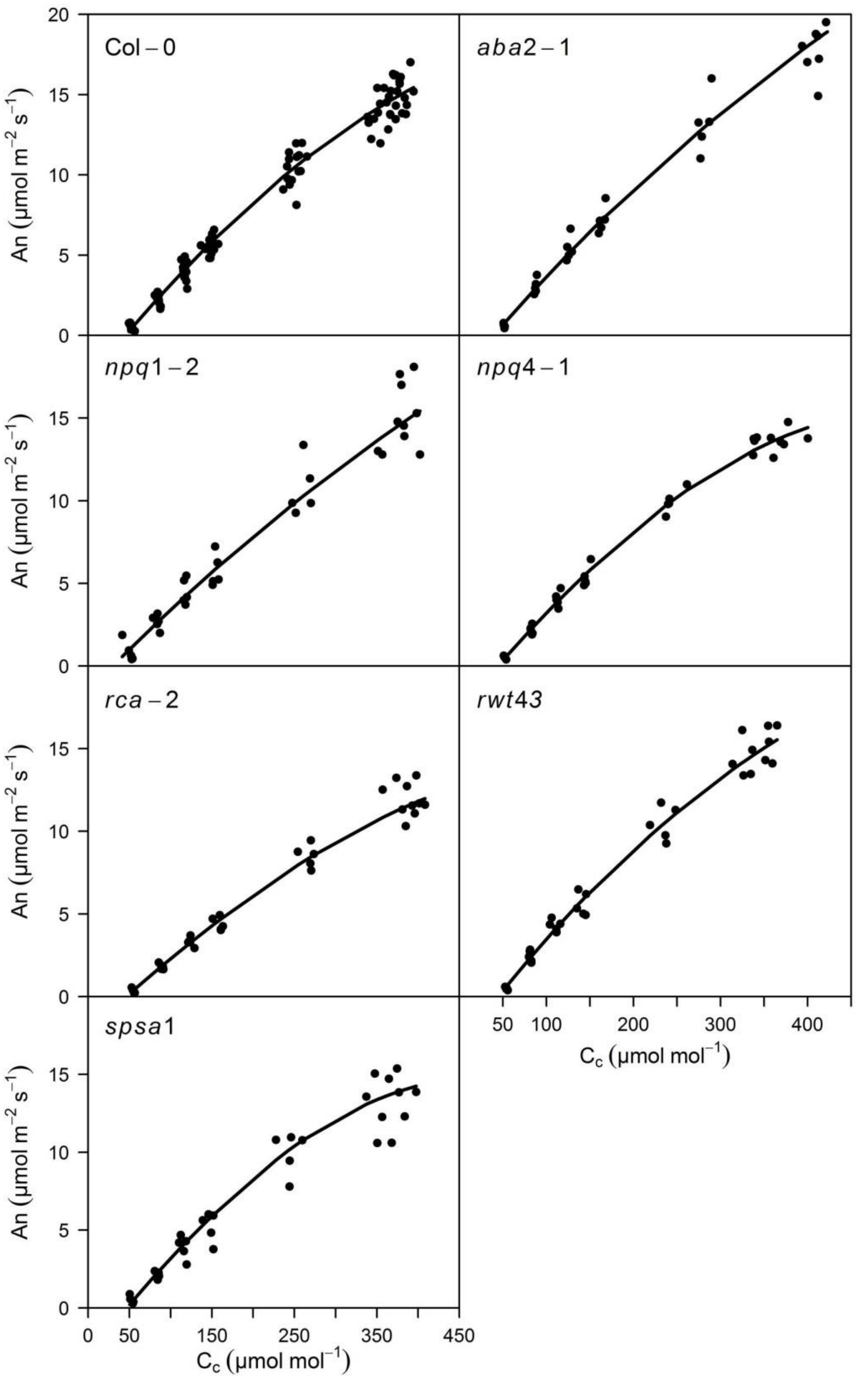

Supplementary Fig. 13. Local polynomial regression fits through data depicting the steady-state A_n/C_c response. The fits were used for correcting the effects of changes in C_i during photosynthetic induction

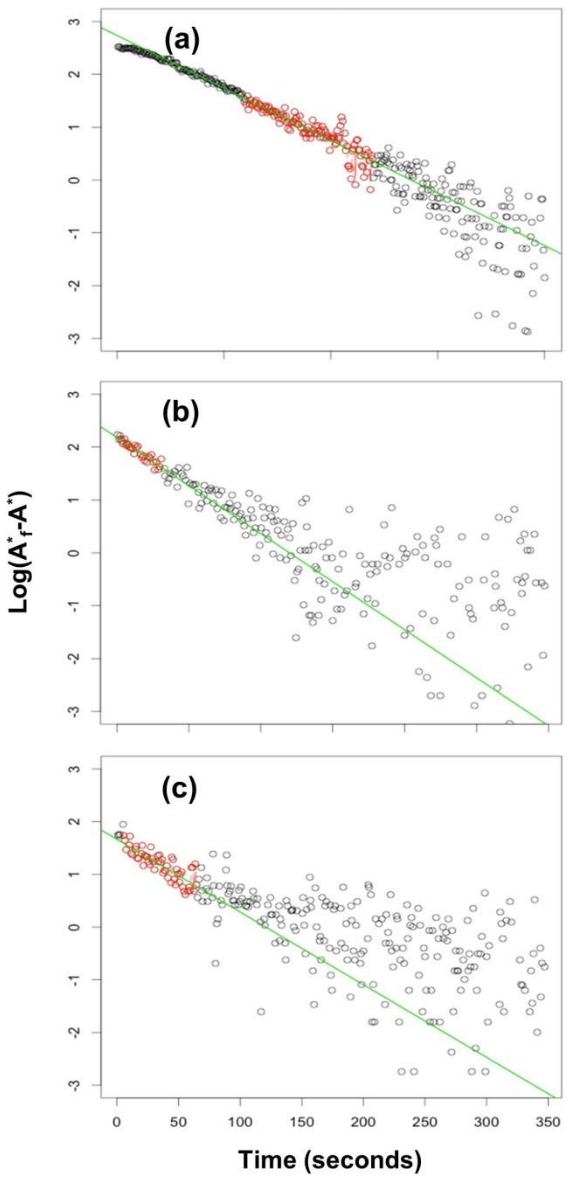

Supplementary Fig. 14. Example of data used to calculate the apparent time constant of Rubisco activation (τ_R) after step increases in irradiance a) 0→1000, b) 70→800 and c) 130→600 µmol m^{-2} s^{-1}. Red dots indicate the data points used to calculate τ_R, which is equal to the inverse of the slope of the green line. Explanation of abbreviations on Y-axis: log, natural logarithm; A^*_f, steady-state net photosynthesis rate at full photosynthetic induction; A^*, transient net photosynthesis rate after irradiance increase, corrected for changes in C_i
